# Single nucleus transcriptomic analysis of rat nucleus accumbens reveals cell type-specific patterns of gene expression associated with volitional morphine intake

**DOI:** 10.1038/s41398-022-02135-1

**Published:** 2022-09-08

**Authors:** Benjamin C. Reiner, Yafang Zhang, Lauren M. Stein, Emilie Dávila Perea, Gabriella Arauco-Shapiro, Jennifer Ben Nathan, Kael Ragnini, Matthew R. Hayes, Thomas N. Ferraro, Wade H. Berrettini, Heath D. Schmidt, Richard C. Crist

**Affiliations:** 1 Department of Psychiatry, Perelman School of Medicine, University of Pennsylvania, Philadelphia, PA USA; 2 Department of Biobehavioral Health Sciences, School of Nursing, University of Pennsylvania, Philadelphia, PA USA; 3 Department of Biomedical Sciences, Cooper Medical School of Rowan University, Camden, NJ USA

**Keywords:** Molecular neuroscience, Addiction

## Abstract

Opioid exposure is known to cause transcriptomic changes in the nucleus accumbens (NAc). However, no studies to date have investigated cell type-specific transcriptomic changes associated with volitional opioid taking. Here, we use single nucleus RNA sequencing (snRNAseq) to comprehensively characterize cell type-specific alterations of the NAc transcriptome in rats self-administering morphine. One cohort of male Brown Norway rats was injected with acute morphine (10 mg/kg, i.p.) or saline. A second cohort of rats was allowed to self-administer intravenous morphine (1.0 mg/kg/infusion) for 10 consecutive days. Each morphine-experienced rat was paired with a yoked saline control rat. snRNAseq libraries were generated from NAc punches and used to identify cell type-specific gene expression changes associated with volitional morphine taking. We identified 1106 differentially expressed genes (DEGs) in the acute morphine group, compared to 2453 DEGs in the morphine self-administration group, across 27 distinct cell clusters. Importantly, we identified 1329 DEGs that were specific to morphine self-administration. DEGs were identified in novel clusters of astrocytes, oligodendrocytes, and D1R- and D2R-expressing medium spiny neurons in the NAc. Cell type-specific DEGs included *Rgs9*, *Celf5*, *Oprm1*, and *Pde10a*. Upregulation of *Rgs9* and *Celf5* in D2R-expressing neurons was validated by RNAscope. Approximately 85% of all oligodendrocyte DEGs, nearly all of which were associated with morphine taking, were identified in two subtypes. Bioinformatic analyses identified cell type-specific upstream regulatory mechanisms of the observed transcriptome alterations and downstream signaling pathways, including both novel and previously identified molecular pathways. These findings show that volitional morphine taking is associated with distinct cell type-specific transcriptomic changes in the rat NAc and highlight specific striatal cell populations and novel molecular substrates that could be targeted to reduce compulsive opioid taking.

## Introduction

Opioid use disorder (OUD) is an ongoing health crisis in the United States for which there is a critical need to understand the molecular basis of compulsive opioid taking [1]. Opioids, including morphine, cause transcriptomic changes throughout the brain. For example, an acute injection of morphine is sufficient to alter the expression of hundreds of genes in the rodent brain [2, 3]. Similarly, repeated, experimenter-delivered morphine has been shown to alter gene expression in multiple brain nuclei, including the nucleus accumbens (NAc) [3–9]. Despite this literature, no studies have investigated cell type-specific transcriptomic changes associated with the escalation of opioid taking, a DSM-5 criteria for diagnosing OUD. Thus, there are significant gaps in our understanding of the molecular mechanisms regulating the transition from acute/recreational opioid taking to compulsive opioid taking.

A notable limitation of gene expression studies to date is the use of bulk lysates. Neural tissue is a heterogenous mix of cell types and bulk transcriptomic approaches lack the resolution to identify changes in gene expression that are specific to individual cell populations. This is particularly problematic in the context of opioids because emerging evidence indicates that repeated opioid exposure induces cell-type-specific effects in the brain. For example, repeated experimenter-delivered morphine injections induce differential gene expression in dopamine receptor type 1 (D1R) and dopamine receptor type 2 (D2R)-expressing medium spiny neurons (MSNs) [10, 11] and astrocytes in the mouse NAc [12]. Thus, there is a critical need to investigate opioid-induced cell type-specific transcriptomic changes to advance our understanding of OUD and identify novel molecular substrates that could be targeted to reduce compulsive opioid taking.

Recent advances in the assay and analysis of neural transcriptomes using single nuclei RNA sequencing (snRNAseq) have provided unprecedented insights into the mRNA landscape of major neural cell types and subpopulations [13–19]. However, no studies have used these approaches to comprehensively characterize cell type-specific gene expression changes in the rat brain following volitional opioid taking. Here, we used snRNAseq to identify cell type-specific alterations of the rat NAc transcriptome after acute morphine exposure and repeated morphine self-administration. We identified cell type-specific differentially expressed genes (DEGs) unique to volitional morphine taking, including gene expression profiles specific to subtypes of astrocytes, oligodendrocytes, and D1R- and D2R-expressing MSNs. We identify cell type-specific affected canonical signaling pathways and gene ontologies, and predicted upstream regulatory mechanisms of the observed volitional morphine taking-induced differential expression. Together, these novel insights into striatal cell-subtype specific mechanisms underlying volitional opioid taking significantly advance our understanding of the molecular basis of OUD.

## Methods and materials

### Animals and housing

Brown Norway rats are an inbred strain, and their genomic homogeneity minimizes the potential for interindividual neural transcriptome differences. Male Brown Norway rats weighing 175–200 g were obtained from Charles River Laboratories (Wilmington, MA, USA). Rats were housed individually with food and water available ad libitum in their home cages. A reverse 12/12 h light/dark cycle was used with the lights on at 1900 h. All experimental procedures were performed during the dark cycle. The experimental protocols were consistent with the guidelines issued by the National Institutes of Health and were approved by the Institutional Animal Care and Use Committee of the University of Pennsylvania.

### Acute morphine exposure

**Fig. 1:**
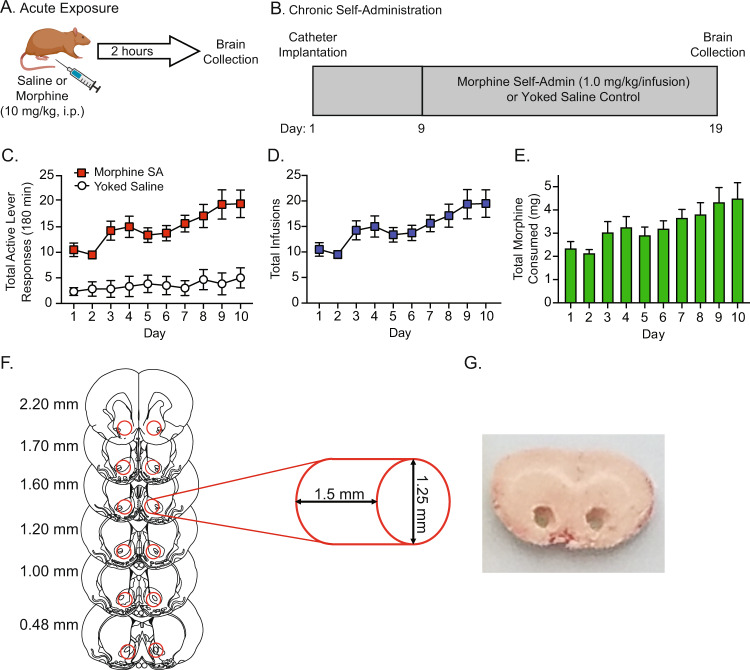
Morphine treatment. Male Brown Norway rats were randomly assigned to the single experimenter-delivered experiment (**A**) or the chronic self-administration (Self-Admin) experiment (**B**). In the acute exposure experiment, rats received a single injection of morphine or saline (*n* = 5 each). In the chronic self-administration experiment, animals were allowed to self-administer morphine in daily test sessions or received yoked saline infusions (*n* = 6 each). The total number of active lever presses (**C**), total number of morphine infusions per day (**D**), and the total morphine infused per day (**E**). Following morphine and saline treatments, brains were frozen and the nucleus accumbens (**F**) was punched from coronal sections (**G**).

Rats were injected with morphine (10.0 mg/kg, i.p.) or saline two weeks after acclimating to their home cages (an age-matched timepoint that coincided with the initiation of morphine self-administration in the second experimental cohorts of rats described below; Fig. 1A). Rats were euthanized two hours post injection. Whole brains were then dissected, flash frozen in −20 °C isopentane, and stored at −80 °C. The dose of morphine and time course of administration were based on previous rodent studies investigating opioid-induced changes in gene expression in discrete brain nuclei [12, 20, 21].

### Morphine self-administration

A separate group of Brown Norway rats was implanted with indwelling jugular catheters as described in our previous intravenous drug self-administration studies (Fig. 1B) [22–24]. Briefly, rats were handled daily and allowed two weeks to acclimate to their home cages upon arrival and grow to the desired weight needed for surgery. Rats were then anesthetized using ketamine (100 mg/kg) and xylazine (10 mg/kg). An indwelling catheter (SAI Infusion Technologies, Lake Villa, IL) was inserted into the right jugular vein and sutured in place. The catheter was routed to a mesh back mount platform that was implanted subcutaneously dorsal to the shoulder blades. To prevent infection and maintain patency, catheters were flushed daily with 0.2 ml of the antibiotic Timentin (0.93 mg/ml; Fisher, Pittsburgh, PA) dissolved in heparinized 0.9% saline (Butler Schein, Dublin, OH). When not in use, catheters were sealed with plastic obturators.

Following 7 days of recovery, the rats were randomly assigned to one of two groups: morphine-experimental or yoked saline controls. Morphine-experimental rats were placed into operant conditioning chambers and allowed to lever-press for intravenous infusions of morphine (1.0 mg/kg/infusion, infused over 5 s) under a fixed-ratio 1 (FR1) schedule of reinforcement similar to our previous studies of opioid self-administration in rats [22, 23]. The unit dose of morphine was based on our pilot studies as well as previous studies of morphine self-administration in rats [25–27]. All self-administration sessions were 3 hours in duration [25–29] and were conducted over 10 consecutive days. Each morphine infusion was paired with a 20 s contingent light cue illuminated directly above the active lever (i.e., drug-paired lever). A 20 s time-out period followed each morphine infusion, during which time active lever responses were tabulated but had no scheduled consequences. Responses made on the inactive lever, which had no scheduled consequences, were also recorded during the self-administration sessions. Each rat that was allowed to respond for contingent morphine infusions was paired with a yoked rat that received infusions of saline. While lever pressing for the yoked saline rats had no scheduled consequences, these rats received the same number and temporal pattern of infusions as self-administered by their paired morphine-experimental rat. Rats were euthanized immediately after their last self-administration session. Whole brains were dissected, flash frozen in −20 °C isopentane, and stored at −80 °C.

### Nuclei isolation, library preparation, and sequencing

Bilateral NAc tissue was punched from frozen brains and nuclei suspensions were prepared, as we described previously (see supplemental methods) [13, 30, 31].

### Quality control and clustering of snRNAseq data

Seurat v3.1 was used to merge read count matrices from all samples. Nuclei with low numbers of detected genes (<539) or a high percentage of mitochondrial transcripts (≥5%) were considered low quality and removed. Nuclei with high numbers of UMIs (>11,365) were also removed to minimize the presence of potential multiplets in the data set. All mitochondrial transcripts were subsequently removed from the remaining data, reflecting the use of nuclei instead of whole cells.

Transcript counts were normalized to 10,000 per nucleus and scaled. Genes with highly variable expression and mean scaled expression between 0.003 and 2 (*n* = 1664) were identified using the mean.var.plot selection method. Principal components (PCs) were generated from these genes and nuclei were clustered in Seurat using the first 50 PCs. Clusters were identified using known cellular markers: Microglia—*Arhgap15*; Endothelial Cells—*Cldn5*, *Ebf1*; Astrocytes—*Gja1*; Oligodendrocyte Precursor Cells—*Pdgfra*; Oligodendrocytes—*Mag*; MSNs—*Bcl11b*, *Drd2*, *Ebf1*, *Grm8*, *Ntng1*, *Nr4a1*; Inhibitory Neurons—*Gad1*, *Sst*, *Kit*, *Kcnc2*; Cholinergic Neurons—*Slc5a7*.

Cell populations with mixed or unclear cell type markers, or with markers (e.g., *Slc17a7*) indicating nuclei of non-NAc origin were removed. A total of 40,223 nuclei were excluded during this step. Normalization, scaling, PC generation, and clustering were redone on the remaining data. An additional three clusters (870 nuclei) were removed due to low numbers of nuclei or lack of representation across samples, for a final total of 190,030 nuclei in 27 distinct cell clusters.

### Identification of DEGs

Count data was converted to log2 counts per million. Differential expression between cases and controls in each cluster was analyzed for the acute injection and chronic self-administration groups separately. The R package MAST [32] was used to fit a linear mixed model with fixed effects for treatment group and the number of genes detected in each nucleus (gdr) and a random effect for subject:

$$\begin{array}{l}{\mathrm{m}} < - {\mathrm{zlm}}\left(\sim{\mathrm{treatment}} + {\mathrm{gdr}} + \left( {\mathrm{1}}\left|{\mathrm{subject}} \right.\right),{\mathrm{sca}},{\mathrm{parallel}} ={\mathrm{TRUE}}, {\mathrm{method}}\right. \\\left.={\hbox{``}}{\mathrm{glmer}}{\hbox{''}},{\mathrm{ebayes}} ={\mathrm{FALSE}},{\mathrm{silent}} = {\mathrm{TRUE}}\right)\end{array}$$

Genes expressed in <20% of the nuclei in a cluster were excluded from the analysis. To optimize the random and fixed effects coefficients in the penalized iteratively reweighted least squares step, the integer scalar in the LME4 R package was set equal to zero, as previously described [33]. Gene expression differences associated with morphine compared to saline were identified with MAST using a Likelihood Ratio Test, which tests for differences between the model with and without the effect of the treatment group. *P* values were corrected for multiple testing using Benjamini-Hochberg correction (FDR = 0.05). Genes with at least a 10% difference in expression between the morphine and saline groups (log2 fold change ≥0.14) and an FDR corrected *p* value < 0.05 were considered differentially expressed.

### Enrichment analyses

As in our prior description [30], the overrepresentation of cell-type-specific DEGs in GWAS phenotypes canonical pathways, gene ontologies, hallmark gene sets, microRNA targets, and transcription factor targets was determined by comparing cluster-specific DEGs, from all clusters with at least 50 DEGs in either the acute or chronic treatment group, from both treatment groups to GWAS Catalog Molecular and Signatures Database (MsigDB) v7.0 [34] using FUMA [35] (see supplemental methods).

### Fluorescent in situ hybridization

To validate select DEGs identified by snRNAseq, a separate cohort of rats was allowed to self-administer intravenous morphine, with yoked saline controls, as described. Fluorescent in situ hybridization was conducted using the RNAscope, similar to our previously published studies [36, 37]. Data are presented as the average integrated density ± SEM (see supplemental methods).

## Results

### Volitional morphine taking

Male Brown Norway rats acquired and maintained robust morphine self-administration (Fig. 1C). Rats escalated their daily intake of morphine over the 10-day self-administration phase (Fig. 1D), similar to published studies from our lab and others investigating voluntary opioid taking in rats [22, 23, 38–40]. Rats self-administered almost double the amount of morphine on day 10 versus day 1 of the self-administration phase, and the total morphine infused was similar to previous rat morphine self-administration studies that used a three-hour daily access paradigm (Fig. 1E) [25, 41, 42]. Micropunches of the NAc (Fig. 1F, G) from the morphine-experienced rats and their respective controls were then processed for snRNAseq (see Table S1 for sample IDs).

### Identification of novel striatal cell populations by single nuclei RNA sequencing

snRNAseq was performed on NAc samples using the 10x Genomics 3' gene expression assay. Nuclei were sequenced to an average depth of ~506 million reads per sample. We initially identified 293,735 putative nuclei, with average medians of ~2063 genes and ~3883 UMI per nucleus (Table S2). The number of sequencing reads per sample, nuclei per sample, average sequencing reads per nuclei per sample, median genes per nuclei per sample, and median UMI per nuclei per sample were consistent between the experimental and control rats in the acute and repeated morphine exposure groups (all Mann–Whitney *U* *p* values > 0.47; Fig. S1 and Table S2). Examination of sequencing-derived surrogates of RNA quality from the CellRanger output data showed no difference in the fraction of reads mapped confidently to the genome, intergenic regions, exonic regions, or the transcriptome between experimental and control rats in both the acute and repeated morphine exposure groups (all Mann–Whitney *U* *p* values > 0.13; Fig. S2 and Table S3). After quality controls (see Methods), 190,030 nuclei remained for downstream analysis.

**Fig. 2:**
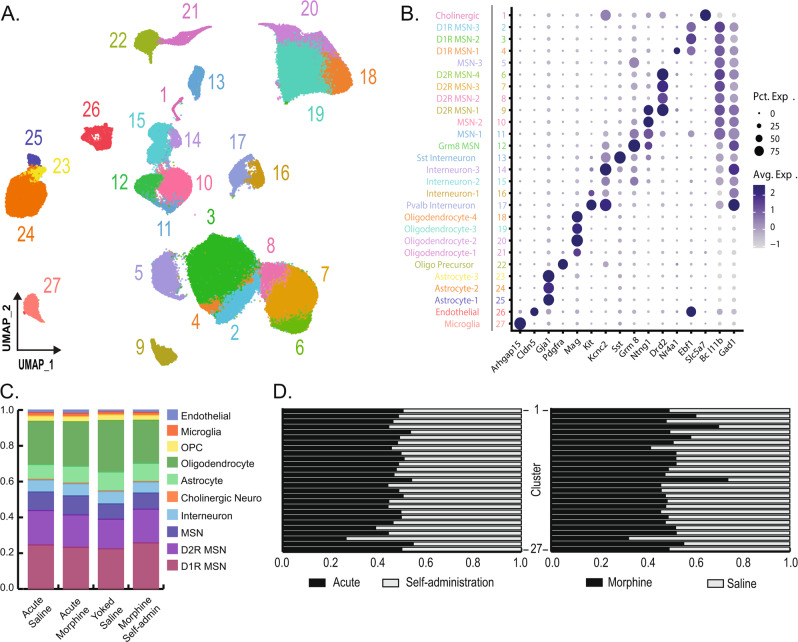
snRNAseq and clustering. **A** After quality controls, ~190,000 nuclei profiles were used for unbiased clustering in Seurat and are presented as a uniform manifold approximation and projection (UMAP) dimension reduction plot of all nuclei color-coded by cluster. **B** Clusters were annotated with genes known to be markers for major neural cell types. The size and color of dots are proportional to the percentage of cells expressing the gene (Pct. Exp.) and the average expression level of the gene (Avg. Exp.), respectively. The cluster numbers and colors are matched to that of the UMAP. **C** The proportion of major cell types in the saline and morphine group for the acute and chronic self-administration (SA) experiment. **D** The proportion of nuclei in each cluster coming from either the acute or chronic self-administration experiment (left) and the morphine or saline group treatment (right). Clusters are in descending order.

Nuclei from all treatment groups were clustered together using Seurat, allowing for direct comparisons of the effects of different morphine exposures on the transcriptome of specific NAc cell types and subpopulations. Clustering with Seurat using principal components derived from variable gene expression identified 27 distinct cell clusters (Fig. 2A), which were then annotated using known expression markers for NAc tissue [43–45] (Fig. 2B). Our data set included all major cell types expected in the NAc and a notable number of D1R- and D2R-expressing MSN sub-populations (Fig. 2B). The number of nuclei from all major cell types were comparable between experimental and control rats in each exposure paradigm (Fig. 2C). Nuclei from all treatment groups were represented in each cluster, in terms of acute vs. self-administration treatments and morphine vs. saline groups (Fig. 2D) and from individual samples (Fig. S3).

### Volitional morphine taking induces unique DEGs

**Fig. 3:**
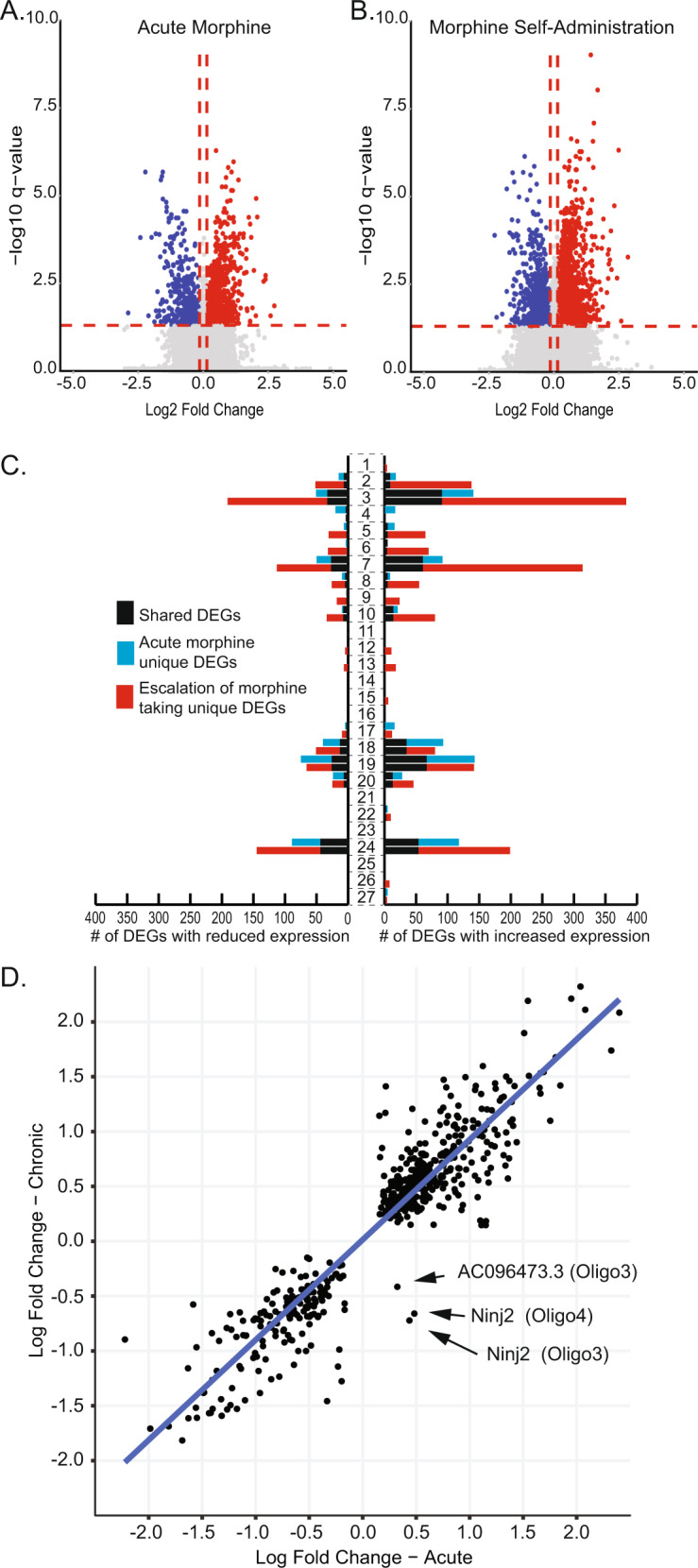
Differential expression. Volcano plots depicting the downregulated (blue) and up regulated (red) differential expressed genes (DEGs) from the acute morphine treatment group (**A**; *n* = 1106 DEGs) or the morphine self-administration (**B**; *n* = 2453 DEGs) experiment. **C** A stacked bar graph depicting the number of downregulated (left) or upregulated (right) shared (black bars) and unique (blue or red bars) DEGs per cluster. **D** Of the 514 differential expression event shared between the acute morphine and morphine self-administration groups, gene expression was altered in the same direction of effect in nearly all instances. Three examples of gene expression alteration changing direction of effect were identified between the acute and self-administration groups.

To identify cell type-specific changes in NAc gene expression following acute morphine exposure and volitional morphine taking, snRNAseq data were analyzed for each cell cluster using a linear mixed model in the *MAST* R package. We found 1106 differential expression events in the acute morphine group (Fig. 3A, Table S4), with cell type-specific DEGs identified in 17 of 27 cell populations. DEGs were 64.6% upregulated and 35.4% were downregulated (Table S5). Similarly, 2,453 DEGs were detected in the morphine self-administration group (Fig. 3B, Table S6), with cell type-specific DEGs in 24 of 27 clusters. DEGs were 67.3% upregulated and 32.7% downregulated (Table S5). Acute morphine exposure and morphine self-administration were associated with a combination of shared and unique DEGs within different cell populations, with cell type-specific differences between upregulated and downregulated genes (Fig. 3C). We identified 514 cell subtype-specific differential expression events shared between the acute morphine and morphine self-administration groups (i.e., a DEG that was detected, in a given cell subtype, in both the acute morphine and morphine self-administration conditions) (Fig. 3C and Table S7). Nearly all shared differential expression events had the same direction of effect on transcript levels between the acute morphine and morphine self-administration treatments, suggesting that these cell subtype-specific transcription alterations were a result of morphine exposure, rather than volitional morphine taking (Fig. 3D). However, there were notable exceptions (Fig. 3D). In the Oligodendrocyte-3 and Oligodendrocyte-4 cell types, *Ninj2* was upregulated after acute morphine exposure, but downregulated after volitional morphine taking. Similarly, *AC096473.3*, a putative isoform of *Galr3*, was upregulated after acute morphine exposure, but downregulated after volitional morphine taking in the Oligodendrocyte-3 cell type. These divergent expression patterns likely represent transcriptional changes associated with repeated morphine taking.

**Fig. 4:**
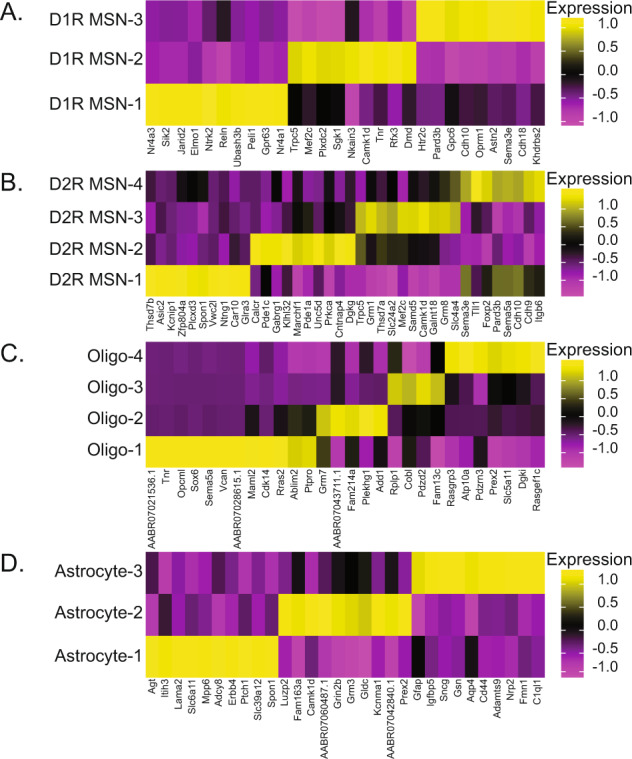
Cell type identification. **A** D1R- and **B** D2R-expressing neuronal cell types are defined by increased expression of unique genes sets. Similarly, **C** oligodendrocyte (Oligo) and **D** astrocyte cell types can be identified by increased expression of unique sets of marker genes.

The majority of DEGs associated with morphine self-administration (54.2%) did not overlap with the acute morphine condition. These cell type-specific transcription alterations included 1,329 differential expression events in 869 unique genes (Table S8). These cell type-specific DEGs unique to volitional morphine taking offer new opportunities to better understand the neurobiological mechanisms underlying morphine self-administration. DEGs unique to volitional morphine taking were not evenly distributed amongst NAc cell types, with some subpopulations accounting for most or all the detected differential expression events within a major cell type (red bars, Fig. 3C; Table S5). To identify DEGs associated specifically with volitional morphine taking and not other aspects of the behavioral procedures (e.g. surgery, repeated handling, stress), we analyzed differential expression between the acute saline and yoked saline control animals. 204 DEGs were identified, with 134 occurring in a single cluster of D1R-expressing MSNs (Table S9). Only a single gene from the saline comparison was also a DEG with the same direction of effect in the morphine self-administration analysis, indicating that the DEGs associated with morphine taking are specifically associated with volitional drug taking. Having observed that differential gene expression patterns specific to volitional morphine taking were associated with distinct striatal cell subtypes, we next sought to define the transcriptome differences that demarcated these cell subtypes (Table S10). In neurons, DEGs specific to self-administration were concentrated in distinct D1R- or D2R-expressing cell subtypes (cell types D1R MSN-2 and D2R MSN-3, respectively; Fig. 4A, B). These neuronal subtypes are clearly defined by distinct patterns of gene expression. In oligodendrocytes, the Oligodendrocyte-4 and Oligodendrocyte-3 subtypes accounted for ~85% of all oligodendrocyte DEGs and nearly all oligodendrocyte DEGs were unique to morphine taking. Like D1R- and D2R-expressing neurons, the oligodendrocyte cell types can be clearly delineated by baseline patterns of gene expression (Fig. 4C). Similarly, the Astrocyte-2 subtype accounted for all astrocytic DEGs and is markedly characterized by a distinct pattern of baseline gene expression (Fig. 4D). Taken together, these studies identify, for the first time, distinct NAc neuronal and glial cell type-specific transcriptomic changes that are associated with volitional morphine taking. Importantly, the identification of these novel NAc cell types, and the transcriptomic markers by which they can be identified in future studies, presents opportunities for further investigating the neurobiological basis of OUD.

### Different molecular mechanisms regulate transcription following acute morphine exposure versus volitional morphine taking

To understand the regulation of the gene expression patterns induced by acute morphine and morphine self-administration, we next sought to identify transcriptional regulatory mechanisms associated with cell subtype-specific patterns in differential expression. We analyzed cell type-specific DEGs lists from the acute morphine treatment group, the morphine self-administration group, the overlap between these phenotypes, and the cell type-specific DEGs unique to the self-administration group. Cell subtype-specific DEG lists for all groups were compared to MSigDB to identify microRNAs (Table S11) and transcription factors (Table S12) whose target genes were statistically overrepresented amongst the cellular subtype-specific DEGs. The cell type-specific microRNA and transcription factors identified for the acute morphine treatment group and the DEGs overlapping both treatment groups are highly similar, suggesting that these transcriptional regulatory mechanisms are invoked in response to the presence of morphine. For example, miR181 and miR141 were identified as regulators of gene expression in neuronal and glial cell types in response to morphine and are linked to the effects of opioids and the regulation of pain [46–50]. Interestingly, the analysis of the DEGs unique to morphine self-administration identified 28 microRNAs and 52 transcription factors as cell type-specific transcriptome regulatory mechanisms that were unique to volitional morphine taking and suggests that volitional morphine taking induces NAc neuronal and glial cellular subtypes to utilize distinct mechanisms for transcription regulation. These results identify direct regulators of transcriptome alterations. To validate the direct regulators and identify indirect upstream regulators of the observed cell type-specific transcriptome alterations, IPA was used to predict activated and inhibited upstream regulatory network master regulators for D1R-expressing MSNs, D2R-expressing MSNs, oligodendrocytes, and astrocytes for the acute morphine and morphine self-administration groups, and for the DEGs unique to the self-administration group (Table S13). Supporting the microRNA and transcription factor analyses, IPA validated many of the cell type-specific direct regulators of transcription, suggesting face validity of these results. Additionally, the analysis identified shared upstream regulators for the acute morphine and morphine self-administration groups, and upstream regulators specific to volitional morphine taking. Taken together, these results identify cell type-specific mechanisms by which volitional morphine taking alters NAc cellular subtype-specific transcriptomes; providing intriguing targets for interventions.

### Volitional morphine intake is associated with cell type-specific neuroadaptations

Observing that acute morphine injection and repeated morphine self-administration altered gene expression in ways that were unique to morphine exposure and volitional morphine taking, we next sought to examine the cell type-specific neurobiological functions affected by these transcriptome alterations. Comparison of cluster-specific DEGs for the acute morphine group, the repeated morphine self-administration group, and the cell type-specific DEGs unique to the self-administration group were conducted. Comparison of cluster-specific DEGs to canonical pathways (Table S14) and gene ontologies (Table S15) identified cell type-specific pathways associated with morphine exposure (i.e., significant in both treatment groups) and pathways unique to volitional morphine taking. For example, the canonical pathways “response to stress”, “response to heat stress”, and “response to external stimuli” were significant in D1R- and D2R-expressing MSNs, oligodendrocyte, and astrocyte clusters in both the acute morphine exposure and repeated morphine self-administration groups, while the canonical pathway “opioid signaling” was significant in a population of D1R-expressing MSNs after morphine self-administration. Additionally, comparison of the cell type specific DEGs to GWAS Catalog identified cell type-specific overrepresentation of genes associated with GWAS phenotypes for both the acute exposure (29 GWAS phenotypes) and repeated self-administration (105 GWAS phenotypes) treatment groups (Table S16). Importantly, morphine self-administration was also associated with cell type-specific GWAS phenotypes (73 GWAS phenotypes) (Table S16). These GWAS phenotypes included “smoking status” and “general risk tolerance”, both of which have been previously associated with substance use disorders. Taken together, these data suggest that the NAc cell subtypes identified in this analysis may represent a portion of the neural circuits associated with these GWAS phenotypes.

### Cell-type-specific validation of target genes in D2R-expressing MSNs

**Fig. 5:**
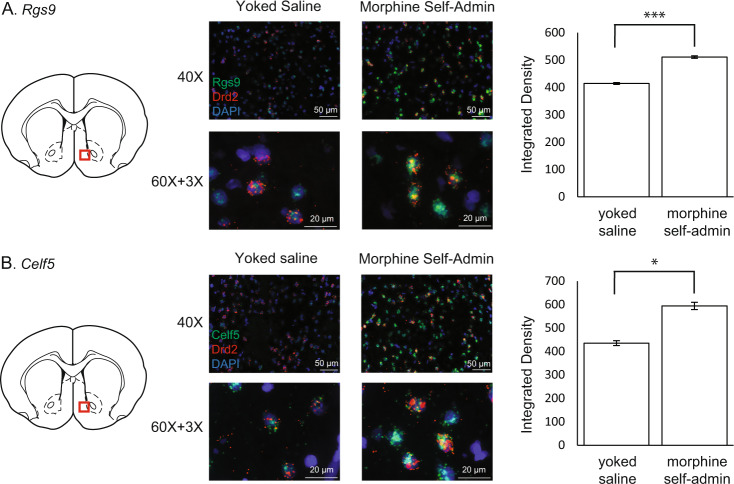
Fluorescence in situ hybridization. RNAscope was used to validate the differential expression of *Rgs9* (**A**) and *Celf5* (**B**) in *Drd2* expressing medium spiny neurons in rats self-administering morphine or their saline yoked controls. Each gene was assessed in four animals (*n* = 4 slices each), and example images for the yoked saline or morphine self-administration are presented. Quantification of integrated florescence density (see Methods) confirmed the increased expression of both genes in *Drd2* expressing neurons of animals that self-administered morphine. *p < 0.05. ***p < 0.001.

To validate cell type-specific differential expression, we utilized florescence in situ hybridization (RNAscope) to quantify changes in the expression of two genes in D2R-expressing MSNs. Given the emerging role of NAc MSN populations in opioid-mediated behaviors [51–53], we identified DEGs specific to morphine self-administration in all four D2R-expressing MSN subtypes. We selected *Rgs9* for validation due to its regulation of mu-opioid receptors and considerable literature linking it to addiction-like phenotypes (reviewed [54, 55]). We also selected a gene with no known role in opioid taking, *Celf5*, the protein product of which is involved in regulating mRNA splicing [56, 57]. NAc slices from rats self-administering morphine and their yoked saline controls (*n* = 4; four slices per rat) were imaged following RNAscope. Quantification of FISH revealed significantly increased expression of *Rgs9* (Control 414.49 ± 3.53; Morphine Self-administration 510.88 ± 4.73; t(30) = 3.9521, *p* < 0.001; Fig. 5A) and *Celf5* (Control 435.43 ± 10.62; Morphine Self-administration 594.25 ± 15.45; t(30) = 2.0504, *p* < 0.05; Fig. 5B) in NAc D2R-expressing MSNs of rats self-administering morphine. These results are consistent with the direction of effect observed in the snRNAseq results.

## Discussion

We used snRNAseq to comprehensively characterize the cell type-specific longitudinal effects of morphine exposure on NAc transcriptomes. We identified novel transcriptomic changes in unique striatal cell populations that are associated with morphine self-administration. These studies represent the first insight into striatal cell type-specific gene expression patterns associated with volitional morphine taking. Furthermore, we were able to identify cell type-specific regulatory mechanisms and affected neurobiological signaling pathways associated with acute morphine exposure or volitional morphine taking. We believe these data are critical to the field, as they provide novel insights into the neural mechanisms associated with a widely employed behavioral model for OUD.

Although there are no prior snRNAseq studies of morphine self-administration for comparison, single-cell transcriptomics in the NAc following an intraperitoneal morphine injection has previously been studied in mice [43]. Our acute morphine data are consistent with the overall trends observed by Avey et al., who found significant gene expression changes primarily in oligodendrocytes, neurons, and astrocytes. Selective transcriptomically distinct subtypes of these three major cell types were the major sources of DEGs in our analysis of acute morphine as well, including the D1R MSN-2, D2R MSN-3, Oligodendrocyte-3, Oligodendrocyte-4, and Astrocyte-2 populations. Critically, these NAc cell types are defined by their transcriptome (Fig. 4), which serves as cell type-specific markers for follow-up studies investigating the functional role of these striatal cell types in opioid-mediated responses. The variance between the cell type-specific DEGs in this study and prior work [43] is likely due to species and technical differences. The pharmacokinetics of opioids differ between mice and rats, including their half-lives, and different time points were applied for analyses between the studies. Additionally, this analysis utilized the 10x Genomics platform for snRNAseq, compared to Drop-Seq [58].

In the current study, the cell types with the most DEGs were similar between acute morphine and morphine self-administration, with astrocytes, oligodendrocytes, and neuronal populations having the largest number of significant changes in gene expression. While cell type-specific gene expression alterations overlapped the treatment groups, suggesting an enduring neurobiological response to morphine, the majority of DEGs in response to self-administration were not shared with the DEGs for investigator-delivered morphine, suggesting that they are gene expression alterations specific to volitional morphine taking. Furthermore, the majority of oligodendrocyte and astrocyte DEGs were concentrated in Oligodendrocyte-3, Oligodendrocyte-4, and Astrocyte-2 cell subtypes, suggesting that these non-neuronal cells shift their gene expression profiles during the transition from acute morphine exposure to repeated morphine taking. Astrocytes have been implicated in opioid addiction [59], with prior evidence showing that morphine alters astrocytic branching [60], and astrocyte regulation of neural maturation [61] and synapse formation [62]. NAc astrocytes have also been shown to regulate morphine-conditioned place preference and synaptic plasticity through morphine-induced alterations of astrocytic metabolic processes [12]. Taken together, these findings suggest that the Astrocyte-2 population may play a role in opioid reinforcement and provide an exciting target for future research. Altered white matter integrity has also been observed in patients with OUDs [63–65] and these changes have been associated with relapse risk [64]. Given the significant transcriptomic changes observed in the Oligodendrocyte-3 and Oligodendrocyte-4 clusters, these specific cell types may be important to this known neuropathological feature of chronic opioid use. Future studies examining additional time points between first exposure and 10 days of self-administration could help better define transcriptomic trajectories in the implicated cell types and explain the specific time frames in which genes and pathways are relevant. The alterations in gene expression associated with volitional morphine taking may include altered genes associated with volitional morphine taking and/or the pharmacological effects of repeated morphine exposure. While a yoked morphine group would have controlled for the number and temporal pattern of repeated intravenous morphine infusion, interpreting results from this potential control group would be limited due to the repeated unpredictable stress associated with yoked morphine infusions. Similarly, while repeated experimenter-delivered injections of morphine avoid the unpredictable stress associated with yoked administration, it poorly recapitulates the pharmacokinetics and temporal pattern of intravenous drug self-administration. Thus, a portion of the DEGs in our dataset may be related to the pharmacological effects of repeated morphine exposure and not volitional morphine taking. Furthermore, the data described here were generated using only male rats and potentially relevant sex differences could not be addressed. Future work should focus on this, as well as the potential role of the estrus cycle.

Morphine self-administration resulted in a greater number of DEGs than acute morphine. Many DEGs associated with morphine self-administration have been implicated in opioid phenotypes. For example, expression of *Oprm1*, the gene encoding the mu-opioid receptor, was downregulated in cluster D2R MSN-2 (cluster #8, Fig. 2A, B). An emerging literature identifies a role for NAc D2R-expressing MSNs in opioid withdrawal [66–68]. Morphine tolerance is regulated by reductions in mu-opioid receptor availability at the cell surface, either by transcriptional regulation or internalization of the receptor [69, 70]. Studies have reported downregulation of *Oprm1* mRNA following chronic experimenter-delivered morphine due to either promoter hypermethylation [71] or an increase in miRNAs that cause degradation of *Oprm1* transcript [72]. Our data suggest that morphine tolerance in the D2R MSN-2 cell type is regulated at the level of transcription, by the downregulation of *Oprm1* transcripts. Whether the morphine-induced downregulation of *Oprm1* in D2R MSN-2 is regulated by promoter epigenetics or miRNA expression remains an open question and an enticing target for future studies. Furthermore, the lack of *Oprm1* gene expression changes amongst the other three D2R-expressing MSN cell types indicates that they may regulate morphine tolerance by internalization of the receptor or that they may not play a morphine tolerance. These potential mechanisms should be further explored in the context of D2R-expressing MSN function and opioid-mediated behaviors.

In contrast to *Oprm1*, *Rgs9* was upregulated in all four D2R-expressing MSN subtypes (clusters #6–9, Fig. 2A, B) following volitional morphine taking. This result was validated in the second cohort of rats (Fig. 5A). A splice variant of *Rgs9*, known as *Rgs9-2*, is highly expressed in the NAc and involved in opioid reward and tolerance [55, 73, 74]. Constitutive Rgs9 knockout alters the rewarding effects of morphine, as well as morphine-induced analgesic responses [55]. Furthermore, optogenetic activation of *Rgs9*+ neurons in the NAc increased development of morphine tolerance [73] and data suggest this tolerance is related to interactions between *Rgs9* and G-protein subunits [75, 76]. Taken together, these data suggest that regulation of *Rgs9* gene expression in NAc D2R-expresssing MSNs is a ubiquitous mechanism of regulating opioid reward and tolerance. *Pde10a* was also upregulated after morphine self-administration in *Sst*+ interneurons (cluster #13) and Astrocyte-2 (cluster #24, Fig. 2A, B). Inhibiting Pde10a has been shown to suppress the acquisition of morphine-conditioned place preference in rats [77]. Pde10a inhibition also facilitated the extinction of conditioned place preference, suggesting that *Pde10a* is involved in morphine-induced learning and memory [77]. These results suggest that inhibiting volitional morphine taking induced an increase of *Pde10a* in the *Sst+* interneuron cell type, the Astrocyte-2 cell type, or both cell types could be a potential future pharmacological target to reduce morphine conditioned responses. In total, the identification of cell type-specific DEGs associated with morphine self-administration in genes previously associated with opioid phenotypes provides face validity for our findings and suggests that the cell type-specific alterations in gene expression represent opportunities for mechanistic study.

The DEGs lists were enriched for large numbers of different GWAS hits, gene ontology (GO) terms, and pathways. While acute morphine exposure and morphine self-administration shared many of these results (e.g., genes related to stress response), there were also distinct findings for volitional morphine taking. For example, cell type-specific microRNA and transcription factors that are putative regulators of DEGs associated with morphine self-administration were identified. Similarly, the GO terms “regulation of glutamate receptor signaling pathway” and “regulation of NMDA receptor activity”, which were driven in part by the presence of *Oprm1* and *Rgs9* in the DEGs of D2R MSN-2 in response to volitional morphine taking. NMDA receptors directly interact with the mu-opioid receptor in the striatum and other brain regions [78]. Furthermore, NMDA receptor antagonists inhibit the development of morphine tolerance without blocking the analgesic effects of acute morphine injection [79]. Studies also suggest NMDA receptor antagonists may be useful in reducing symptoms associated with opioid withdrawal [80–82]. These previous findings fit well with the results of the present study and support the idea that alterations to NMDA receptor activity occur in the context of volitional morphine taking, but not acute morphine exposure.

In conclusion, we identified unique cell type-specific changes in the NAc transcriptome that were associated with volitional morphine taking. These DEGs may represent possible cell type-specific molecular targets for novel therapeutic approaches to treating OUD. Future studies investigating the functional significance of these genes will be required to understand the therapeutic potential of these targets. Future snRNAseq studies can also extend our findings in a number of directions. Analyses of tissue from rats self-administering different opioids may reveal differences in cell type-specific effects between common opioids of abuse. Studying single nuclei transcriptomics during morphine withdrawal and the reinstatement of opioid seeking, a model of relapse, will also expand our understanding of the longitudinal transcriptomic changes that promote relapse during acute and prolonged withdraw.

## Supplementary information

Supplemental Materials

Supplemental Tables

## Data Availability

Single-nuclei RNA-sequencing data are available at the NCBI Gene Expression Omnibus (GEO) under accession number GSE171165.
