# Supplementary material for: Single nucleus transcriptomic analysis of rat nucleus accumbens reveals cell type-specific patterns of gene expression associated with volitional morphine intake
Source: Transl Psychiatry. 2022 Sep 8;12:374. doi: 10.1038/s41398-022-02135-1 (PMC9458645; doi:10.1038/s41398-022-02135-1)
Supplement: Supplementary file 1 — Supplemental Materials [file 41398_2022_2135_MOESM1_ESM.docx]

**Supplemental Methods and Materials**

Nuclei Isolation, Library Preparation, and Sequencing

Bilateral NAc tissue was punched from frozen brains and nuclei suspensions were prepared, as described previously (1-3). Briefly, NAc tissue punches were homogenized in 500 µL lysis buffer (Nuclease-free water with 10 mM Tris-HCL, 10 mM NaCl, 3 mM MgCl_2_, 0.5% NP-40), diluted with an additional 2.5mL of lysis buffer, incubated for 5 minutes on ice, and then mixed with 3mL of wash buffer (1X PBS with 2% BSA, 1:1000 RNase inhibitor, 0.25% Glycerol). To remove debris, samples were passed through a 30μm cell strainer, pelleted by centrifugation, and resuspended in wash buffer a total of three times. Nuclei were counted using Trypan Blue and resuspended in wash buffer at a concentration of 1,000 nuclei/µL.

Single nuclei RNA-seq libraries were generated at the Children’s Hospital of Philadelphia Center for Applied Genomics (CAG) using the 10x Genomics Chromium microfluidics platform. 20,000 nuclei per sample were loaded onto the Chromium controller, to target a capture of ~10,000 nuclei. Sequencing libraries were produced with the 3’ Gene Expression Kit v3.1 according to manufacturer’s instructions. Libraries for all samples were pooled at equimolar concentrations and sequenced on a single NovaSeq 6000 S4 flow cell at CAG. Sequencing data were demultiplexed and aligned to the pre-mRNA reference rat transcriptome (Rnor6.0.101) using CellRanger v3.1.0.

Fluorescent *In situ* hybridization

To validate select DEGs identified by snRNAseq, a separate cohort of rats was allowed to self-administer intravenous morphine for 10 consecutive days on a FR1 schedule of reinforcement. Each morphine-experienced rat was paired with a yoked saline control rat as described above. Rats were sacrificed and whole brains dissected immediately following the last self-administration session. Brains were stored at -80°C until processed. Coronal sections (8 µm) were taken at the level of the striatum using a cryostat (Leica 3050S; Leica Corp., Deerfield, IL). Fluorescent *in situ* hybridization was conducted using the RNAscope multiplex fluorescent reagent kit v2 (323100, Advanced Cell Diagnostics, Newark, CA) similar to our previously published studies (4, 5). Briefly, brain sections were incubated in 10% neutral buffered formalin at 4°C for 15 min. Sections were then dehydrated in 50%, 70%, 100% and 100% ethanol at room temperature in 5 min consecutive washes. Following dehydration, sections were incubated in hydrogen peroxide for 10 min and protease IV for 30 min at room temperature. Sections were then incubated in the following probes at 40°C for 2 hrs: Rn-Drd2-C3 (315641-C3), Rn-Rgs9 (869661), Rn-Celf5 (1063271-C1). Following signal amplification, opal dyes were applied to the sections. Opal dyes were diluted in TSA buffer (1:1000): Opal 520 reagent (OP-001001, Akoya, Marlborough, MA) and Opal 570 reagent (OP-001003, Akoya, Marlborough, MA). Following hybridization, sections were coverslipped using Fluoro-Gel II mounting medium with DAPI (Electron Microscopy Sciences; Hatfield, PA). Sections were then visualized with a Keyence fluorescence microscope using 40x and 60x oil-immersion objectives with a step size of 1.0 μm. Images were analyzed using the BZ-X800 analyzer (Keyence; Itasca, IL). To quantify *Rgs9* and *Celf5* expression in *Drd2*-expressing cells, images of the NAc were taken using the 40x objective at four levels relative to bregma (+1.70, +1.60, +1.20, and +1.00 mm A/P). For quantification, images were stitched to 1.631 mm x 1.223 mm for +1.70 and +1.60 mm A/P sections, and 1.377 mm x 1.033 mm for +1.20 and +1.00 mm A/P sections. Image sizes and exposure times were consistent across all experiments.

Image processing and quantification was performed by a researcher blinded to experimental treatments. For each rat (n = 4 per treatment), four sections were converted to 8-bit and a binary nucleus mask was created from the DAPI channel. Images were smoothed by dilating and filling intensity holes. Segmentation of neighboring nuclei was carried out via watershed (ImageJ, Version 2.1.0). The open-source ImageJ plugin EZColocalization was used to determine colocalization of the reporter (*Drd2*) and target (*Rgs9* and *Celf5*) genes. A default thresholding was applied to the DAPI mask and an Otsu automated threshold algorithm was applied to the reporter and gene of interest images. The three channels were aligned and colocalization was determined based on a threshold overlap score (TOS) with linear scaling (6, 7). The metric threshold was set at 10% of top pixels for signal intensity. Any ROI with no colocalization or a score of 1.0 (indicating 100% colocalization within that cell, which we deemed likely to be an artifact) was excluded, and any ROI with an area less than 0.01 pixels or greater than 1.0 pixels, based on outlier analysis for all ROI area size, was excluded. Finally, integrated density was calculated as a measurement of fluorescent signal intensity on ROIs with both reporter and target gene expression. Data are presented as the average integrated density ± SEM and were analyzed using an unpaired t-test in SPSS (v24).

Enrichment Analyses

The overrepresentation of cell type specific DEGs in GWAS phenotypes was determined by comparing cluster-specific DEGs from both treatment groups to GWAS Catalog using FUMA (8). Similarly, identification of canonical pathways, gene ontologies, hallmark gene sets, microRNA targets, and transcription factor targets that were significantly overrepresented in cluster-specific DEGs was analyzed using FUMA and data from the Molecular and Signatures Database (MsigDB) v7.0 (9). For all analyses, all clusters with at least 50 DEGs in either the acute or chronic treatment group were analyzed and all genes, except the MHC region, from Ensembl v92 were used for the gene background. All results were corrected for multiple testing using a Bonferroni correction (α=0.05). Upstream regulator analysis was conducted using Ingenuity Pathway Analysis (10). P-values were calculated using a right-tailed Fisher’s exact test and corrected for multiple testing using a Benjamini-Hochberg correction (α=0.1).

**Supplemental Figures**

**Supplementary Figure 1**: **Sequencing statistics**. snRNAseq was performed on male Brown Norway rat NAc following acute morphine injection, 10-day morphine self-administration, or saline controls (acute injection or yoked saline, respectively) using a the 10x Genomics Chromium platform and 3’ gene expression assay. Sequencing data was initially analyzed using the 10x Genomics Cell Ranger pipeline (see Methods). (**A**) The number of sequencing reads per sample did not vary between cases and controls in each treatment group (Acute Control 508,238,762.8 ± 33,594,283.7; Acute Morphine 485,407,618.0 ± 44,221,284.3; Acute Mann-Whitney U = 11, z = 0.21, p = 0.83366; Self-administration (SA) Control 509,330,175.5 ± 12,816,384.7; SA Morphine 517,181,463.7 ± 20,925,060.4; SA Mann-Whitney U = 16, z = -0.24, p = 0.81034). (**B**) The number of nuclei identified per sample (Acute Control 14,427.4 ± 1,305.2; Acute Morphine 13,528.0 ± 1,715.7; Acute Mann-Whitney U = 9, z = -1.00, p = 0.31732; SA Control 12,466.0 ± 2,082.1; SA Morphine 13,193.8 ± 1,451.6; SA Mann-Whitney U = 15, z = -0.40, p = 0.68916) and (**C**) the average number of sequencing reads per nuclei per sample (Acute Control 35,736.0 ± 5,825.4; Acute Morphine 36,774.0 ± 7,061.8; Acute Mann-Whitney U = 10, z = -0.82, p = 0.41222; SA Control 42,223.8 ± 8,339.3; SA Morphine 39,769.5 ± 5,395.9; SA Mann-Whitney U = 16, z = 0.24, p = 0.81034) did not differ between cases and control in each treatment group. Nuclei from each treatment group did not differ in (**D**) the median number of genes detected per nuclei (Acute Control 2,070.0 ± 125.0; Acute Morphine 2,066.0 ± 195.0; Acute Mann-Whitney U = 9, z = -1.00, p = 0.31732; SA Control 2,017.3 ± 144.3; SA Morphine 2,099.8 ± 173.6; SA Mann-Whitney U = 13, z = -0.72, p = 0.47152) or (**E**) the median number of UMI per nuclei (Acute Control 3,901.0 ± 291.7; Acute Morphine 3,835.0 ± 497.3; Acute Mann-Whitney U = 8, z = -1.19, p = 0.23404; SA Control 3,823.0 ± 323.1; SA Morphine 3,968.5 ± 487.8; SA Mann-Whitney U = 15, z = -0.40, p = 0.68916). Data presented as Mean ± Standard Deviation. Box and Whisker plots display the median (center line), first and third quartiles (box), and the maximum and minimum values within 1.5x the interquartile distance (whiskers). Circles denote data points in addition to maximum and minimum whiskers.


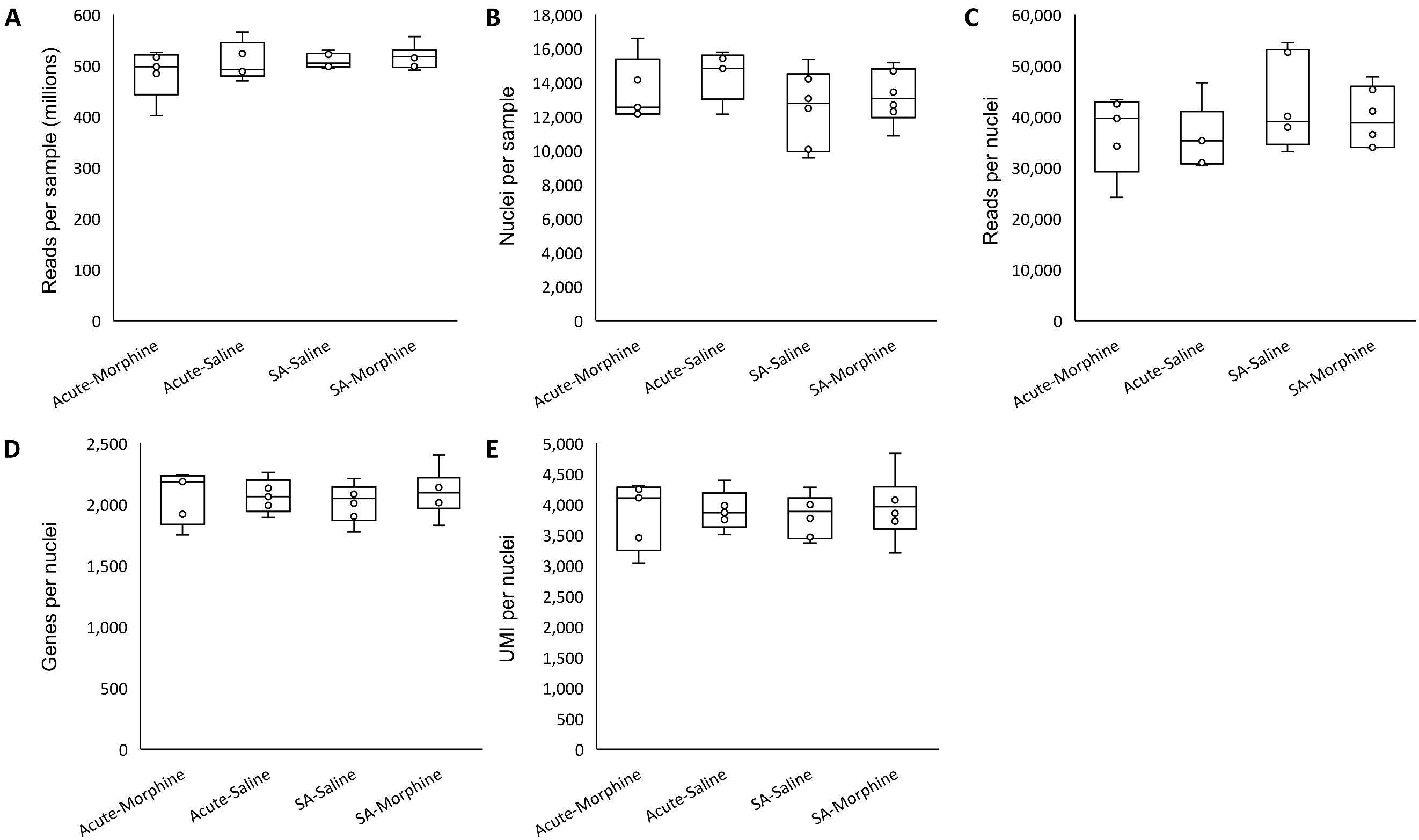


**Supplementary Figure 2**: **RNA quality**. To ensure that RNA quality was consistent between samples, we investigated sequencing derived surrogates of RNA quality. There was no difference in the fraction of reads mapped confidently to the (**A**) genome (Acute Control 0.8674 ± 0.0033; Acute Morphine 0.8666 ± 0.0038; Acute Mann-Whitney U = 10, z = 0.42, p = 0.67448; Self-administration (SA) Control 0.8698 ± 0.0039; SA Morphine 0.8690 ± 0.0033; SA Mann-Whitney U = 14.5, z = 0.48, p = 0.63122), (**B**) intergenic regions (Acute Control 0.2360 ± 0.0099; Acute Morphine 0.2356 ± 0.0058; Acute Mann-Whitney U = 11, z = -0.21, p = 0.83366; SA Control 0.2343 ± 0.0041; SA Morphine 0.2422 ± 0.0118; SA Mann-Whitney U = 13, z = -0.72, p = 0.47152), (**C**) exonic regions (Acute Control 0.6312 ± 0.0114; Acute Morphine 0.6312 ± 0.0092; Acute Mann-Whitney U = 10.5, z = 0.31, p = 0.75656; SA Control 0.6362 ± 0.0061; SA Morphine 0.6268 ± 0.0129; SA Mann-Whitney U = 8, z = 1.52, p = 0.12852), or (**D**) the transcriptome (Acute Control 0.4992 ± 0.0184; Acute Morphine 0.4994 ± 0.0196; Acute Mann-Whitney U = 11, z = -0.21, p = 0.83366; SA Control 0.5067 ± 0.0145; SA Morphine 0.4943 ± 0.0215; SA Mann-Whitney U = 8, z = 1.52, p = 0.12852) between the cases and control in each treatment group. Data presented as Mean ± Standard Deviation. Box and Whisker plots display the median (center line), first and third quartiles (box), and the maximum and minimum values within 1.5x the interquartile distance (whiskers). Circles denote data points in addition to maximum and minimum whiskers.


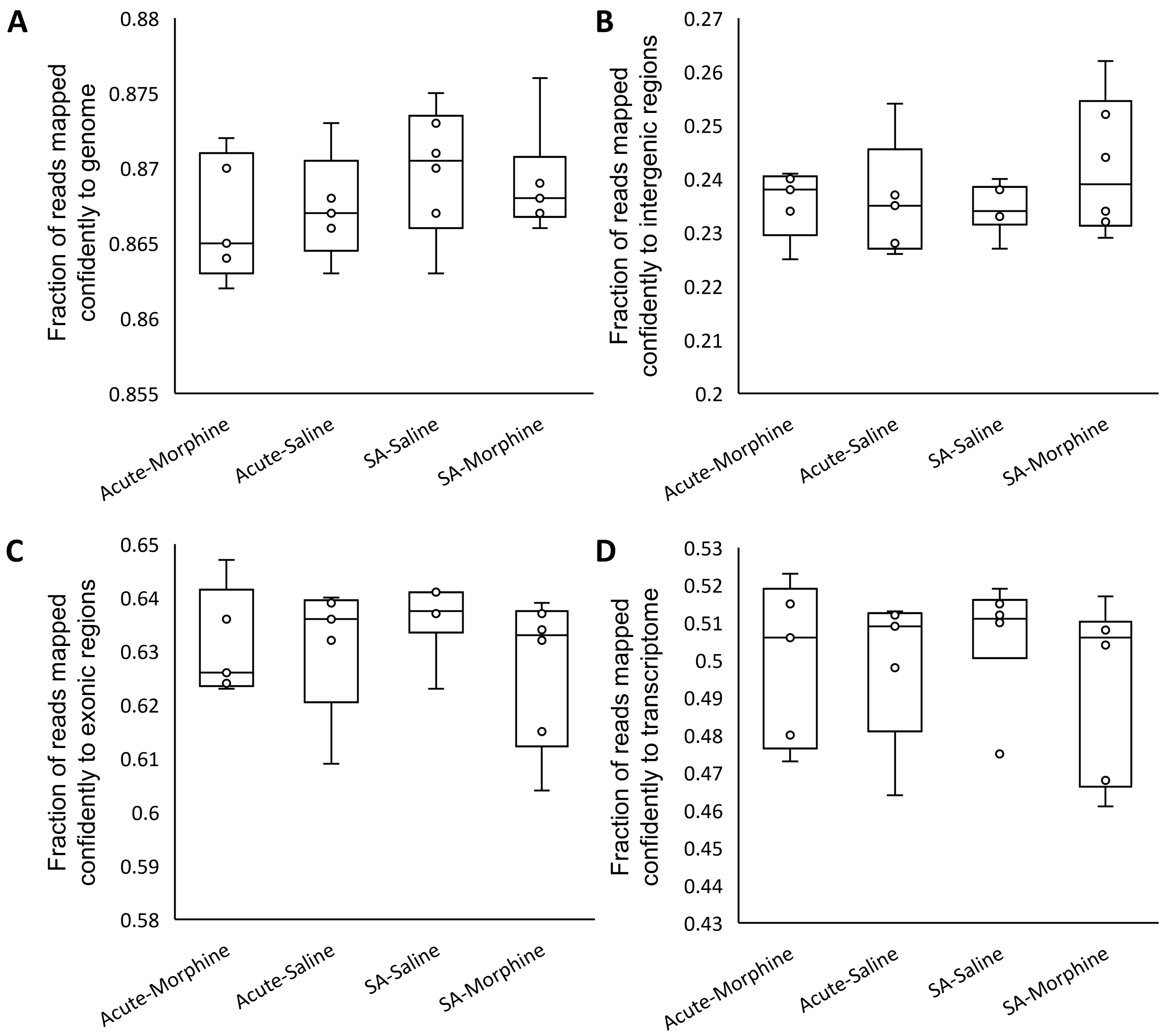


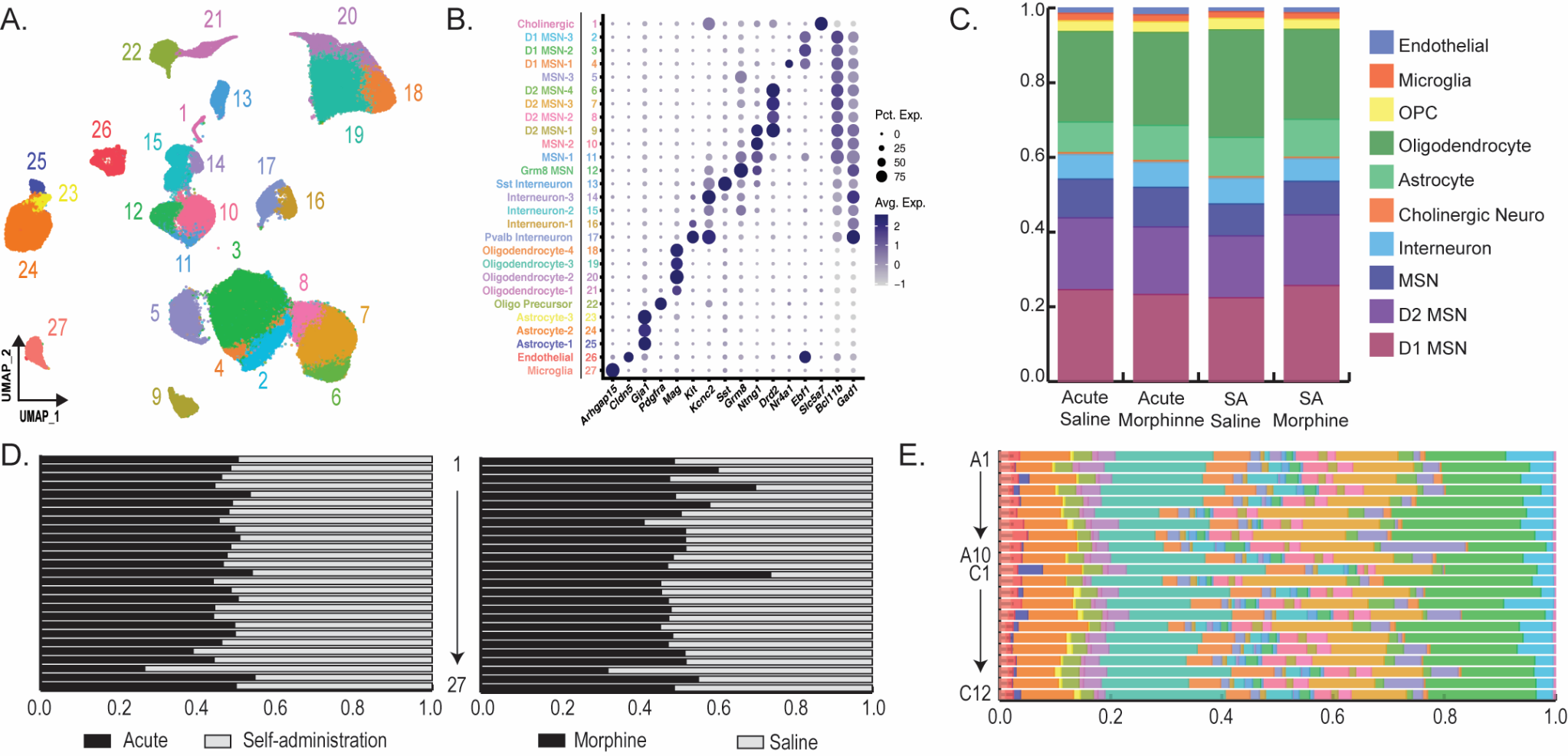


**Supplementary Figure 3**: **Nuclei per sample**. A bar graph depicting the proportion of nuclei from each sample contributing to each cluster. Sample numbers correspond to those in the supplementary tables and color coding of clusters correspond to Figure 2A-B. As expected, while the absolute number of post-QC nuclei from each sample in each clusters does vary, similar proportions are seen throughout the samples.

1. Borner T, Geisler CE, Fortin SM, Cosgrove R, Alsina-Fernandez JA, Dogra M, et al. (2021): GIP Receptor Agonism Attenuates GLP-1 Receptor Agonist Induced Nausea and Emesis in Preclinical Models. *Diabetes*.

2. Reiner BC, Crist RC, Borner T, Doyle RP, Hayes MR, De Jonghe BC (2021): Single nuclei RNA sequencing of the rat AP and NTS following GDF15 treatment. *Molecular Metabolism*. in press.

3. Reiner BC, Crist RC, Stein LM, Weller AE, Doyle GA, Arauco-Shapiro G, et al. (2020): Single-nuclei transcriptomics of schizophrenia prefrontal cortex primarily implicates neuronal subtypes. *BioRxiv*.

4. Hernandez NS, Weir VR, Ragnini K, Merkel R, Zhang Y, Mace K, et al. (2020): GLP-1 receptor signaling in the laterodorsal tegmental nucleus attenuates cocaine seeking by activating GABAergic circuits that project to the VTA. *Molecular Psychiatry*.

5. Fortin SM, Lipsky RK, Lhamo R, Chen J, Kim E, Borner T, et al. (2020): GABA neurons in the nucleus tractus solitarius express GLP-1 receptors and mediate anorectic effects of liraglutide in rats. *Sci Transl Med*. 12.

6. Sheng H, Stauffer WT, Hussein R, Lin C, Lim HN (2017): Nucleoid and cytoplasmic localization of small RNAs in Escherichia coli. *Nucleic acids research*. 45:2919-2934.

7. Sheng H, Stauffer W, Lim HN (2016): Systematic and general method for quantifying localization in microscopy images. *Biol Open*. 5:1882-1893.

8. Watanabe K, Taskesen E, van Bochoven A, Posthuma D (2017): Functional mapping and annotation of genetic associations with FUMA. *Nat Commun*. 8:1826.

9. Liberzon A, Subramanian A, Pinchback R, Thorvaldsdottir H, Tamayo P, Mesirov JP (2011): Molecular signatures database (MSigDB) 3.0. *Bioinformatics*. 27:1739-1740.

10. Kramer A, Green J, Pollard J, Jr., Tugendreich S (2014): Causal analysis approaches in Ingenuity Pathway Analysis. *Bioinformatics*. 30:523-530.
